# The Prevalence of Climacturia in Patients after Radical Prostatectomy: A Systematic Review

**DOI:** 10.1590/S1677-5538.IBJU.2024.0406

**Published:** 2025-01-10

**Authors:** João Vítor Ferrão, Alice Scalzilli Becker, Gustavo Konopka, Gustavo Bangemann, Thiago Oliboni, Nilson Marquardt, Carlos Teodósio Da Ros, Gustavo Franco Carvalhal

**Affiliations:** 1 Pontifícia Universidade Católica do Rio Grande do Sul Faculdade de Medicina Porto Alegre RS Brasil Faculdade de Medicina, Pontifícia Universidade Católica do Rio Grande do Sul - PUCRS, Porto Alegre, RS, Brasil; 2 Pontifícia Universidade Católica do Rio Grande do Sul Departamento de Urologia Porto Alegre RS Brasil Departamento de Urologia, Pontifícia Universidade Católica do Rio Grande do Sul - PUCRS, Porto Alegre, RS, Brasil; 3 Universidade Luterana do Brasil Disciplina de Urologia Canoas RS Brasil Disciplina de Urologia, Universidade Luterana do Brasil – ULBRA, Canoas, RS, Brasil

**Keywords:** Prostatic Neoplasms, Prostatectomy, Systematic Review [Publication Type]

## Abstract

**Purpose:**

Prostate Cancer (PCa) is the most common non-cutaneous cancer in males, and Radical Prostatectomy (RP) is among the primary treatments for this condition. Our study aims to investigate the prevalence of climacturia (urine leakage at the moment of the climax), a potential post-RP change related to orgasm.

**Material and Methods:**

A systematic review was conducted following PRISMA guidelines and registered on the PROSPERO platform. The search was performed using MEDLINE via PubMed.

**Results:**

Thirteen studies met the inclusion criteria and were described separately. Within these studies, 5,208 patients were evaluated, among which 1,417 cases of climacturia were identified, with a prevalence of 27.2%. When we analyzed the robot-assisted radical prostatectomy (RARP) subgroup, the prevalence of climacturia was 5.7% vs 1.8% the open radical prostatectomy (ORP) subgroup.

**Conclusion:**

Climacturia is a frequently underestimated complication by urologists. Given its significant impact on quality of life, it warrants greater attention from specialists following RP.

## INTRODUCTION

Prostate cancer (PCa) is the most common non-cutaneous cancer in males, with an approximate annual prevalence in 2024 of 299,010 cases in the U.S (1) In Brazil, PCa is also frequent, with an annual prevalence in 2023 of 71,730 cases, which represents 30% of all malignant neoplasms in males (2). In addition, PCa is the second most common cause of death in the male population, and it is expected that approximately 1 in 8 men will have this type of cancer diagnosed during their lives (3). Despite PCa having a high frequency among men, in most cases the cancer is localized at diagnosis and one of the most common treatments for these cases is radical prostatectomy (RP) (4).

In RP the entire prostate gland and the seminal vesicles are removed, and the surgery can be performed using three different approaches: the first and more traditional method is the open prostatectomy; the second is the laparoscopic prostatectomy; and the third is the robotic-assisted radical prostatectomy, in which the laparoscopic surgery is done assisted by robotic arms. Among the many possible complications of RP, erectile dysfunction, changes in orgasm and urinary incontinence are the most frequent (4). However, these complications seem to have neither a statistical difference when analyzing the techniques, the robotic platforms disposable in RARP or between ethnic groups (5-7). Nonetheless, minimally invasive surgery has best perioperative and complication outcomes (8).

Climacturia is one of the possible changes related to orgasm and pleasure that may occur after RP. It refers to a patient who notices various amounts of urine leakage at the moment of the climax (9). Research on this condition is relatively new: the first study to address orgasm-associated incontinence after RP was performed by Koeman et al. in 1996 (10); and the term climacturia was first coined by Lee et al. in 2006 (9). Prevalence of climacturia is still uncertain and variable. Several studies have reported rates ranging from 20% to 93% following RP (11, 12).

Climacturia is associated with a worse quality of life, as it can be bothersome and may lead to patient's avoiding sexual activity due to the embarrassment of urine leakage (11). While various series have evaluated the prevalence of climacturia, only two evaluated the prevalence of patient bother, with 44-48% of patients reporting significant bother and 21% perceiving a significant concern by their partners (12), but we know that the level of discomfort must be much greater but it simply has not yet been correctly assessed.

However, the pathophysiology of climacturia after RP is yet to be determined. Various mechanisms have been suggested, but none have been adequately tested (13). It is likely that anatomical alterations following RP, such as a decrease in functional urethral length, nerve damage and trauma to the bladder neck or urethral sphincter play a pivotal role (11, 14).

To the present moment, climacturia does not have a well-defined prevalence and probably has not been reported as adequately as it should, even though it is bothersome and impacts patients’ quality of life. This study aims to understand the prevalence of this common complication of RP, increasing the awareness about this situation and pointing at possible solutions to this condition, through a systematic review of the literature.

## MATERIALS AND METHODS

This systematic review was conducted according to PRISMA guidelines (15) and registered on the PROSPERO platform (PROSPERO no. CRD42021279827).

### Search strategy

The research question was performed using the PICO method. We included patients who underwent RP, regardless of the surgical technique. Intervention was surgical treatment and the comparison was whether, or not, patients reported climacturia after RP. The main outcome variable was the prevalence of the climacturia in all patients analyzed.

We systematically searched for climacturia as a post-RP complication in the MEDLINE via PubMed database, from January 2016 until May 2024. The search terms used included the following: "radical prostatectomy" OR "post-radical prostatectomy" OR "post-prostatectomy" [MeSH] AND "climactur*" OR "Orgasm-Associated Incontinence" OR "Orgasm-associated urinary incontinence" [MeSH] OR "sexual incontinence" OR "arousal incontinence" OR "coital incontinence" OR "complicat*" [MeSH]. The term "complication" was restricted to the title, as we sought to filter studies that aim to directly analyze the complications of RP. Inclusion criteria were journal articles published in the last eight years (2016-2024) in English, which presented the prevalence or incidence of the climacturia after RP.

Studies were included in the Rayyan platform (16) for recording decisions. The search and selection process adhered to the requirements of PRISMA guidelines (15).

**Figure 1:**
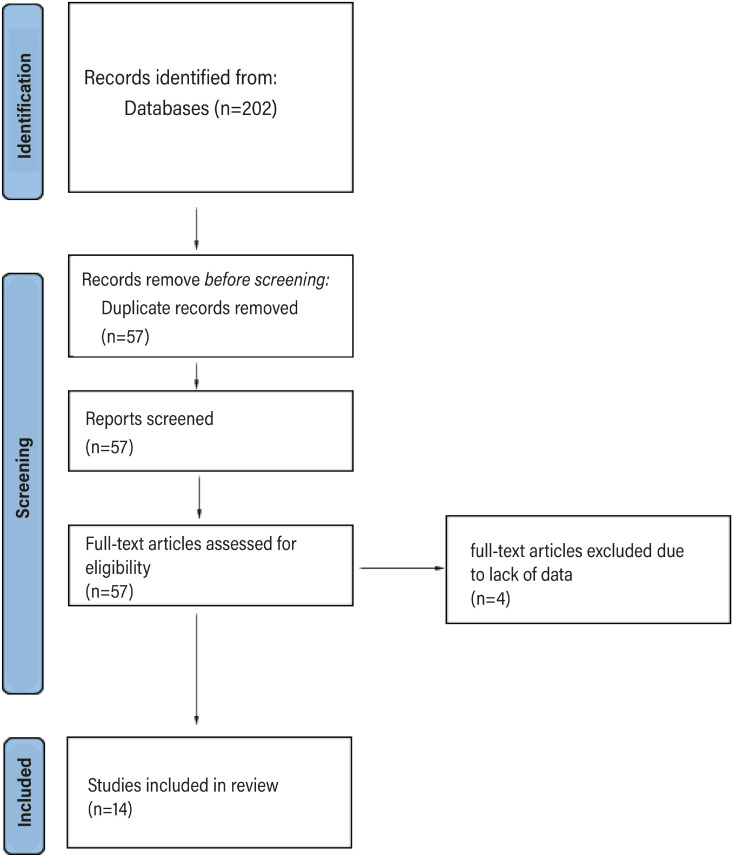
Flow chart summarizing the studies selection process.

Prospective studies, retrospective studies, observational cohorts and case series reporting the prevalence of climacturia were included. We excluded case reports and articles that referred to the same series of patients. In these circumstances, we included the most recent series from the same group. Gray literature, such as congress presentations, meeting abstracts, posters, etc., was not included in the study. Figure-1 summarizes the flow diagram of study screening and selection.

### Data extraction and quality assessment

Data collected from each manuscript included: author names, year of publication, country, study design, analytic sample size and prevalence of climacturia.

Two authors were responsible for selecting the articles. They dually and independently reviewed titles and abstracts, before conducting a full-text review of all potentially eligible studies. At each stage, decisions were compared and discrepancies resolved by consulting a third researcher. An Excel® spreadsheet was used for record decisions.

We analyzed the quality of the non-randomized studies using the Newcastle-Ottawa Quality Assessment Scale (NOS), more precisely the scale for Case Control Trials. In this method we evaluated studies with eight different items that were divided into three sections (Selection, Comparability and Exposure). The studies could range from 0-4 points in the Selection section, from 0-2 points in the Comparability section, and from 0-3 points in the Exposure section. NOS scale assigns scores range from one to nine, with research scoring less than four points are considered to be of low quality. Quality assessment of the included studies is presented in Table-1 (17).

**Table 1:** Quality assessment of the studies using NOS scale.

Study	Newcastle-Ottawa Scale			
	Selection	Comparability	Exposure	Score
Capogrosso et al. (2016) (18)	★★	★★	★★★	7
Yafi et al. (2018) (19)	★★★	★★	★★★	8
Andrianne (2019) (20)	★★	★	★★	5
Salter et al. (2019) (12)	★★	★★	★★	5
Towe et al. (2019) (21)	★★	★	★	4
Valenzuela et al. (2019) (22)	★★	★	★★	5
Jimbo et al. (2020) (23)	★★	★★	★★	6
Nolan et al. (2020) (24)	★★	★	★★	5
Sullivan et al. (2020) (25)	★★	★★	★★★	7
Parra López et al. (2021) (26)	★★★	★★	★★★	8
Hammad et al. (2023) (28)	★★	★★	★	5
Honda et al. (2022)(27)	★★★★	★★	★★★	9
Huynh et al. (2023) (29)	★★★★	★★	★★★	9
Gamberini et al. (2024) (30)	★★★	★	★★	6

## RESULTS

The search strategy resulted in 202 articles. Of these, 144 were excluded after we analyzed their titles and abstracts. Of the remaining 57 articles, four did not have their full text found due to lack of data. After the full text analysis of the 54 remaining articles, 14 studies comprising 5,186 patients were included (Figure-1). Table-2 summarizes the data extracted from each manuscript.

**Table 2:** Individual characteristics of studies included in the systematic review.

Author (year)	Country	Study design	Analytic sample size	Surgical approach	Overall climacturia	Climacturia by procedure
Capogrosso et al. (2016) (18)	Italy	Retrospective study	749	395 (ORP) 354 (RARP)	221	94 (ORP) 127 (RARP)
Yafi et al. (2018) (19)	USA	Prospective pilot study	38 (36/38 RP)	16 (ORP) 20 (RARP)	30	NA
Andrianne (2019) (20)	Belgium	Retrospective study	15	NA	6	NA
Salter et al. (2019) (12)	USA	Retrospective study	3207	1122 (ORP) 770 (LP) 1315 (RARP)	745	NA
Towe et al. (2019) (21)	USA	Case series	2	2 (RARP)	2	2 (RARP)
Valenzuela et al. (2019) (22)	USA	Retrospective chart review	36	NA	30	NA
Jimbo et al. (2020) (23)	USA	Retrospective study	RP alone (139), RT alone (22), RP + RT (31)	NA	73	54 (RP alone), 3 (RT alone), 16 (RP+RT)
Nolan et al. (2020) (24)	USA	Retrospective chart review	17	NA	11	NA
Sullivan et al. (2020) (25)	USA	Retrospective study	194	NA	56	NA
Parra López et al. (2021) (26)	Spain	Retrospective study	62	NA	11	NA
Honda et al. (2022)(27)	Japan	Retrospective study	259	259 (RARP)	44	44 (RARP)
Hammad et al. (2023) (28)	USA	Retrospective study	38	10 (ORP) 11 (LP or RARP)	21 (remaining cohort)	NA
Huynh et al. (2023) (29)	USA	Retrospective study	339	339 (RARP)	127	127 (RARP)
Gamberini et al. (2024) (30)	Brazil	Retrospective study	60	46 (ORP) 11 (LRP) 3 (RARP)	40	NA

RP = radical prostatectomy; RT = radiation therapy; RARP = robot-assisted radical prostatectomy; ORP = open radical prostatectomy; NA = not available

From fourteen studies which met the criteria, twelve (12, 18-20, 22-29) were retrospective reviews, while one was a case series of two subjects (21) and one was a prospective pilot study (19). A total of 5,208 urological procedures were related to these studies, resulting in 1,417 cases of climacturia with different patterns which, in most cases, were not even discussed due to a different focus of the articles. The overall prevalence of 27.2% represents that over a quarter of the individuals have episodes of arousal incontinence at the climax after RP.

Regarding the prevalence of climacturia by procedure, only four studies distinguish whose patients were submitted to ORP or RARP. When we analyze the RARP subgroup, the prevalence of climacturia was 5.7% vs 1.8% the ORP subgroup.

## DISCUSSION

Although the well-known complications of RP, such as erectile dysfunction and stress urinary incontinence, are actively investigated postoperatively and have a wide range of treatments available, other complaints, such as changes in orgasm and climacturia, have not received much attention. However, it has an important impact on quality of life. The literature, until now, does not bring a precise prevalence of climacturia among the studies. Our study aims to address this gap in the literature by presenting an overall prevalence of climacturia at 27.3% across the studies examined. In addition, when performing a subgroup analysis, we could demonstrate that patients submitted to ORP had 1.8% vs 5.7% of those who underwent RARP. Nonetheless, this data does not highlight the real subgroup prevalence, due to the lack of data between the studies selected.

The majority of studies that were found in the initial search, before the selection process, didn't include the prevalence of climacturia. Although, even with the low number of articles eligible to the review, two articles (12, 18) were important in defining the scenario of climacturia in recent years.

Even if some hypotheses are raised (25), there is no consensus about the pathophysiology of climacturia. Koeman et al. were the first to propose that climacturia is a direct side effect of damage to the internal sphincter during RP. This incontinence would probably be caused by the complete absence of the patient's internal sphincter, combined with the "normal" relaxation of the sphincter at the time of orgasm (10). This hypothesis was weakened as other studies suggested a possible correlation between external sphincter injury and internal sphincter dysregulation, eventually causing climacturia (31). On the other hand, O’Neil et al. reported climacturia in 5.2% of patients treated with radiation as radical therapy for PCa (32). This supports the hypothesis that radiation-induced nerve damage can lead to the development of climacturia. In addition, attached to the theory of nerve damage, during surgery the indirect stretching of the pudendal nerve can disrupt the physiological mechanism of ejaculation, thus leading to functional dyssynergia (33).

Capogrosso et al. collected answers of 749 patients from a non-validated 28-item questionnaire. Predominantly, 395 (52.7%) patients were treated with Open Radical Prostatectomy (ORP) and 354 (47.3%) with Robot Assisted Radical Prostatectomy. The groups didn't differ in postoperative outcomes. Furthermore, 221 patients (29.5%) reported postoperative climacturia. It was evoked as occurring at every orgasm by 42 patients (19%) and described as occurring more than half of the time by 31 patients (14.1%). Self-reported volume of orgasm-associated urine leakage was ≥ 5mL in 85.2% of the patients. Interestingly, robotic-assisted RP was correlated with faster climacturia recovery compared to open RP (18).

In a prospective, multicenter, pilot study of 38 patients that presented with climacturia and/or mild urinary incontinence (two or less pads per day) post-RP, the patients underwent inflatable penile prosthesis insertion with concomitant placement of a mini-jupette graft. Data were collected in the US, France, Belgium, Germany and Korea. Of the 38 patients who underwent the mini-jupette sling procedure, 30 had post-RP climacturia. Additionally, after the procedure, climacturia showed improvement in 22 of 28 (78.6%) patients with follow-up, of which 19 (67.9%) had complete resolution (19).

Andrianne, in a cohort study, described post-RP climacturia with significant psychological suffering in six of fifteen patients (incidence of 40.0%). between 2006 and 2015 (20).

Salter et al. performed a retrospective analysis at a single center of patients who presented for the management of postoperative sexual dysfunction between 2006 and 2018, who had previously undergone RP. A total of 3,207 patients post-RP were included in the analysis. The mean age was 61±7 years. Most (97%) were heterosexual and 82% were Caucasian. The median time between the procedure and survey was 203 days. Forty-one percent of men had a RARP, 35% had an ORP and 24% underwent a laparoscopic prostatectomy. Men with climacturia were slightly younger (60.3 years old) than men without climacturia (61.6 years old). Forty-five percent of the men with climacturia admitted to being bothered by their symptom, 62% reported mild bother, 29% moderate and 9% severe bother. Of them, 745 (23%) experienced climacturia post-RP: 70% reported a small volume of urine leakage (drops) whereas 24% reported moderate volume (<30mL) and only 6% had large volume (>= 30mL). Thirty-one percent of men categorized climacturia as rare, 47% as occasional, and 22% as frequent (12).

Towe et al. reported a case series that documented two patients who presented with concomitant erectile dysfunction and climacturia following RP, and who subsequently underwent IPP surgery with placement of an autologous fascial mini jupette sling (21).

Valenzuela et al. aimed to describe a technique modified by Yafi et al of the Mini-Jupette sling with placement of an inflatable penile prosthesis (IPP) (19). They followed 36 patients during postoperative clinic visits and with telephone calls. From those, 30 patients (83%) reported climacturia (22).

Jimbo et al. developed an 89-item questionnaire for all patients who presented to their Men's Health Clinic between 2014 and 2017. A total of 1,359 patients fulfilled the intake questionnaire. Of these, 1,117 (82%) reported that they were able to achieve orgasm and 192 (17%) patients had a prior history of definitive therapy for PCa: 139 (72%) underwent RP alone, 22 (11%) had a history radiotherapy (RT) alone and 31 (16%) underwent RP and RT. Among the 139 patients with a history of RP, 54 (39%) reported climacturia and among the 31 patients who underwent RP and RT, 16 (52%) reported the same problem. Overall, 60/170 patients (35%) undergoing RP or RT+RP reported climacturia. Additionally, 26 (15.2%) of those who performed RP or RP+RT felt some level of discomfort. Finally, among the 925 patients with no prior history of prostate cancer or RP, 22 (2.4%) reported climacturia, of which ten (45%) said it was uncomfortable for them (23).

Nolan et al. sent 42 patients a questionnaire that was revised from Lee et al (9). They aimed to assess patients’ changes in climacturia complaints after implantation of a urethral sling as a treatment for stress incontinence after RP. From those patients, 17 were returned for analysis. The median age of the sample at RP was 64 years. At the time of the study, 11 (64.7%) reported continued urinary loss during sexual arousal (24).

Sullivan et al. analyzed retrospectively a database of their sexual medicine database. A total of 194 patients were included in the analysis, 138 patients without and 56 (28.8%) with climacturia. The mean age of patients that reported climacturia were 59±7 years. Applying a multivariate model of logistic regression, urethral width was associated with climacturia (OR 1.34, 95% CI 1.05-1.71, p=0.02) (25).

Parra Lopez et al. conducted a retrospective study of 100 patients that underwent RARP. After excluding patients who didn't meet the eligibility, they were left with 62 patients who were investigated about presence and intensity of climacturia, orgasm quality, incontinence and erectile dysfunction. The mean age of patients with climacturia was 56 years. Eleven patients (17,9%) reported climacturia. Of those, 82% reported slight leaks and 18% reported severe leaks. In 37% of these patients, climacturia occurred in all orgasms (26).

Honda et al. found 417 eligible patients from 523 patients who underwent RARP. They excluded patients who underwent neoadjuvant therapy, adjuvant therapy, or died of other causes. They collected answers of 259 patients with a median age of 67 years old. Overall, 145 (56%) patients were sexually active after surgery; of those, 44 patients (30.3%) reported climacturia, with 39 (88.6%) losing a small amount of urine, four (9.1%) losing a moderate amount, and one (2.3%) losing a large amount. Furthermore, they performed a multivariate logistic regression analysis to identify predictors and found urinary incontinence (OR 3.13, 95% CI 1.20-8.15) as a significantly predictive factor of climacturia (27).

Huynh et al. aimed to primarily measure the incidence and risk factors for climacturia and penile length shortening following RARP. Eight-hundred patients were retrospectively surveyed, with 339 (42%) and 369 (46%) responding about climacturia and penile length shortening. Among these patients, 127 (37.5%) reported climacturia. And this complaint was related to the absence of bilateral nerve sparing (29).

A recent study published by Gamberini et al. retrospectively surveyed sixty patients and actively investigated the occurrence of orgasmic disorders. The authors reported climacturia as the most common orgasmic disorder in 40 (66.6%) patients. However, only 14 patients (35%) reported that it frequently occurs more than half of the time. Among the patients who reported climacturia, 72.5% classified it as mild losses (30).

Moreover, several conservative and invasive treatments have been proposed for climacturia. For instance, behavioral treatment (such as emptying the bladder before sexual arousal and using a condom), pelvic floor muscle training, and the use of external occlusion loops (28, 34, 35). For patients with persistent bothersome climacturia, surgical interventions are sometimes required. The Mini-Jupette procedure (an inflatable penile prosthesis and a sling) has shown that it makes the climacturia disappear in 82% of patients who presented with erectile dysfunction and climacturia (20). The ideal material used as graft still needs to be determined. Valenzuela et al. published an approach called "Male Urethral Mini-Sling". Their modification to the Andrianne's "Mini-Jupette" was based on the proximal placement of a modified sling (Virtue™ mesh from Coloplast), leading to the complete resolution of climacturia and of stress urinary incontinence in 93% of patients (22).

Our systematic review has some limitations - we have accessed only one database (Pubmed), and included only literature in English language, which may have left out of our review other relevant publications. Our data is based on the validity and analysis of our selected published studies. In addition, the majority of the studies found are of low methodological quality. Most of them are retrospective and only two are of reliable quality. Finally, further studies about climacturia should be conducted. The creation of some sort of questionnaire that could be used by all patients of RP describing the frequency, duration and other characteristics of the climacturia would be a useful tool to evaluate, prevent and treat this common manifestation that may occur after radical prostatectomy.

## CONCLUSION

Our review demonstrated an average prevalence of 27.3% of climacturia in patients undergoing different surgical techniques to treat PCa. Climacturia is underestimated by urologists, due to the lack of medical knowledge and the lack of a more targeted assessment through validated questionnaires, for example. This is a very relevant complication, as quality of life and sexuality are greatly compromised. Prospective studies are needed to determine the most appropriate approaches to managing this relatively common complication after radical prostatectomy.
